# Influence of the SARS-CoV-2 Outbreak on the Uptake of a Popular Smoking Cessation App in UK Smokers: Interrupted Time Series Analysis

**DOI:** 10.2196/19494

**Published:** 2020-06-11

**Authors:** Olga Perski, Aleksandra Herbeć, Lion Shahab, Jamie Brown

**Affiliations:** 1 Department of Behavioural Science and Health University College London London United Kingdom; 2 Department of Clinical, Educational and Health Psychology University College London London United Kingdom

**Keywords:** SARS-CoV-2, COVID-19, smoking cessation, mobile health, smartphone app, time series analysis, smoking, public health, app

## Abstract

**Background:**

The severe acute respiratory syndrome coronavirus 2 (SARS-CoV-2) outbreak may motivate smokers to attempt to stop in greater numbers. However, given the temporary closure of UK stop smoking services and vape shops, smokers attempting to quit may instead seek out mobile health support, such as smartphone apps.

**Objective:**

We examined, using an interrupted time series approach, whether the SARS-CoV-2 outbreak has been associated with a step change or increasing trend in UK downloads of an otherwise popular smoking cessation app, Smoke Free.

**Methods:**

Data were from daily and nondaily adult smokers in the United Kingdom who had downloaded the Smoke Free app between January 1, 2020, and March 31, 2020 (primary analysis), and January 1, 2019, and March 31, 2020 (secondary analysis). The outcome variable was the number of downloads aggregated at the 12-hourly (primary analysis) or daily level (secondary analysis). The explanatory variable was the start of the SARS-CoV-2 outbreak, operationalized as March 1, 2020 (primary analysis), and January 15, 2020 (secondary analysis). Generalized additive mixed models adjusted for relevant covariates were fitted.

**Results:**

Data were collected on 45,105 (primary analysis) and 119,881 (secondary analysis) users. In both analyses, there was no evidence for a step change or increasing trend in downloads attributable to the start of the SARS-CoV-2 outbreak. Calculation of Bayes factors (BFs) indicated that the data for the primary analysis favored the null hypothesis compared with large associations (for level, BF=0.25; for slope, BF=0.26) but were insensitive to the detection of small associations (for level, BF=0.78; for slope, BF=1.35).

**Conclusions:**

In the United Kingdom, between January 1, 2020, and March 31, 2020, and between January 1, 2019, and March 31, 2020, there was no evidence that the SARS-CoV-2 outbreak has been associated with a large step change or increasing trend in downloads of a popular smoking cessation app. Findings on the association of the SARS-CoV-2 outbreak with a small step change or increasing trend were inconclusive.

## Introduction

The global outbreak of severe acute respiratory syndrome coronavirus 2 (SARS-CoV-2), which causes the coronavirus disease (COVID-19) [1], was imported into the United Kingdom in January 2020 [2]. Community transmission has subsequently been established [3]. The growing spread of SARS-CoV-2 has been accompanied by a series of government announcements of escalating social distancing policies and legislation. On March 8, 2020, prime minister Boris Johnson unveiled the UK Coronavirus Action Plan [4], which was followed by government advice to practice social distancing on March 16 and behavioral restrictions enforceable by law on March 23, 2020.

Definitive evidence on whether current smokers are at increased risk of disease, morbidity, and mortality from COVID-19 are not yet available. However, researchers and clinicians have emphasized biological (eg, reduced respiratory immune defense) and behavioral (eg, repetitive hand-to-mouth movements) factors that may mean that smokers are at an increased risk of contracting SARS-CoV-2 [5-7], and early evidence indicates that hospitalized smokers may be more likely to experience complications and mortality from COVID-19 compared with nonsmokers [8,9]. UK public health messaging focused on advice to stop smoking has been limited. On March 20, 2020, the charitable organization Action on Smoking and Health launched an online stop smoking campaign labelled “Today Is The Day” [10] with an accompanying hashtag, #QuitForCovid (see Table 1 for a timeline of key UK events related to smoking and COVID-19). On April 3, 2020, Public Health England released a news story headlined “Smokers at greater risk of severe respiratory disease from COVID-19” [11], which generated substantial coverage [12,13].

**Table 1:** Timeline of key UK events in 2020 related to smoking and COVID-19, including government policies and legislation [14].

Date	Key event
January 31	First two cases reported in the United Kingdom
February 7	Primary care guidance updated to state that the virus is most likely to be seen in travelers returning from China, Hong Kong, Japan, Macau, Malaysia, Republic of Korea, Singapore, Taiwan, or Thailand
February 21	At least 17 temporary GP^a^ closures occur
February 24	4 UK passengers repatriated from a Japanese cruise ship test positive for COVID-19^b^, bringing the total number of UK cases to 13
March 2	GP practice closures continue
March 6	First UK patient dies from COVID-19
March 8	Prime Minister Boris Johnson unveils the Coronavirus Action Plan
March 11	World Health Organization declares the severe acute respiratory syndrome coronavirus 2 outbreak a pandemic
March 12	UK COVID-19 risk level raised from moderate to high
March 16	UK government advises on social distancing (eg, avoiding nonessential travel and contact with others); pregnant women, adults 70 years or older, and individuals with health conditions are urged to self-isolate
March 18	UK government announces that schools will close to most students, with children of key workers and vulnerable children still able to attend
March 20	Restaurants, pubs, clubs, and indoor sport and leisure centers ordered to close; Action on Smoking and Health launches the stop smoking campaign “Today Is The Day” and #QuitForCovid
March 23	UK government announces restrictions made on freedom of movement, enforceable by law
April 3	Public Health England releases a news story emphasizing the increased risk of severe respiratory disease due to COVID-19 in smokers

^a^GP: general practice.

^b^COVID-19: coronavirus disease.

Options for UK smokers interested in quitting usually include specialist stop smoking services and vape shops. However, the introduction of social distancing policies and behavioral restrictions enforceable by law mean that the vast majority of services and shops have temporarily closed. At the same time, it is plausible that the SARS-CoV-2 outbreak has acted as a “teachable moment,” motivating smokers to attempt to stop in greater numbers than would otherwise be observed for this time of the year. Given these temporary closures of stop smoking services and vape shops, smokers attempting to quit during the SARS-CoV-2 outbreak may have to seek out digital support, such as websites and smartphone apps. If so, it would be important for public health bodies to put resources toward ramping up access to high-quality, evidence-informed digital support.

One way of assessing whether the recent SARS-CoV-2 outbreak has been accompanied by a surge in smokers accessing digital support is to use an interrupted time series approach to examine whether there has been a step change or increasing trend in downloads of an otherwise popular smoking cessation app. The Smoke Free app, designed for English speakers, includes behavior change techniques that can improve the chances of quitting and shows early evidence of effectiveness [15]. The app is live on the Apple and Google Play stores and has a large user base with >1 million global downloads per annum. The app exists as a free version with a set of core features and a paid (“pro”) version with additional content.

This study used an interrupted time series approach to assess whether the SARS-CoV-2 outbreak up to the end of March 2020 (ie, a hypothesized “teachable moment”) has been accompanied by an increase in UK downloads of the free and “pro” versions of the Smoke Free app (henceforth referred to as the Smoke Free app). Two different starting points for the outbreak were used. First, we assessed whether there has been a step change or increasing trend (linear or nonlinear) in downloads during a period of government announcements of escalating social distancing policies and legislation, with March 1, 2020, as the stipulated starting point (see Table 1; a series of government announcements occurred throughout March 2020). Second, we assessed whether there has been an increasing trend (linear or nonlinear) in downloads over the course of the SARS-CoV-2 outbreak so far, compared with the preceding 12 months, with January 15, 2020, as the stipulated starting point. The middle of January 2020 was selected as a starting point to reduce confounding by the regularly observed annual spike in downloads from late December to mid-January.

Specifically, we aimed to address the following research questions:

1. Over a 3-month time series (ie, January 1, 2020, to March 31, 2020), has a period of government announcements of escalating social distancing policies and legislation (ie, March 1, 2020, to March 31, 2020) been accompanied by a step change or increasing trend in UK downloads of the Smoke Free app?
2. Over a 15-month time series (ie, January 1, 2019, to March 31, 2020), has the SARS-CoV-2 outbreak thus far (ie, January 15, 2020, to March 31, 2020) been accompanied by an increasing trend in UK downloads of the Smoke Free app?

## Methods

### Study Design

This was a natural experiment without active recruitment. The study protocol and analysis plan were preregistered on the Open Science Framework [16]. The study involved the analysis of anonymized app data at the aggregate level and fell within the scope of “The optimisation and implementation of interventions to change behaviours related to health and the environment,” approved by University College London’s Research Ethics Committee (Project ID: CEHP/2020/579). Users provide consent for their data to be used in research by virtue of downloading the app and agreeing to the terms and conditions.

### Study Setting and Population

To be included in the analytic sample, daily and nondaily smokers needed to be 18 years or older and have downloaded the Smoke Free app from a UK-based app store (ie, Google Play and Apple App Store) during the respective study periods.

### Measures

#### Outcomes

For the primary analysis, the outcome of interest was the number of UK Smoke Free app downloads aggregated at the 12-hourly level. This temporal unit was selected to increase the number of observations in the postintervention period.

For the secondary analysis, the outcome of interest was the number of UK Smoke Free app downloads aggregated at the daily level.

#### Exposures

For the primary analysis, the 60-day period (or 120 observations) before the government announcements of escalating social distancing policies and legislation was coded as 0, and the 31-day period (or 62 observations) containing the announcements was coded as 1. The trend during the 31-day postintervention period from March 1, 2020, to March 31, 2020, (or 62 observations) was coded from 1-62.

For the secondary analysis, the 379-day period (or 379 observations) before the start of the SARS-CoV-2 outbreak was coded as 0, and the trend over the 77-day period containing the outbreak from January 15, 2020, to March 31, 2020, (77 observations) was coded from 1-77.

#### Covariates

For the primary analysis, the time of day was included as a categorical covariate (midnight to 11:59 AM or “morning” vs noon to 11:59 PM or “evening”), and the day of the week was included as a cyclic cubic spline to capture nonlinear, seasonal patterns in downloads.

For the secondary analysis, the month of the year and the day of the week were included as cyclic cubic splines to capture nonlinear, seasonal patterns in downloads. Two additional covariates were included. On December 19, 2019, a new expert feature, which involved the ability to communicate directly with stop smoking counsellors, was launched for “pro” users (ie, “expert feature launch”). On December 27, 2019, a promotional offer to purchase a separate version of the app called Smoke Free Plus in a range of Boots stores across the United Kingdom was announced (ie, “national advertising campaign”). The periods before and after the expert feature launch and the national advertising campaign were coded as 0 and 1, respectively.

### Data Analysis

The analyses were conducted in R v.3.6.3 (R Foundation for Statistical Computing) using the *mgcv* package*.*

For the primary analysis, an interrupted time series analysis (segmented regression) was conducted using a generalized additive mixed model (GAMM). GAMMs take account of seasonality through the fitting of seasonal smoothing terms [17]. First, data were assessed for overdispersion (ie, when the variance is greater than the mean). As there was evidence for overdispersion, a quasi-Poisson distribution was specified. Second, plots of the autocorrelation function and partial autocorrelation function were assessed to identify plausible values for the autoregressive (AR) and moving average (MA) terms. Third, a segmented regression model was fitted. Different models with plausible AR and MA terms were compared using the Akaike information criterion (AIC), with smaller values indicating better model fit. The final segmented regression model included terms for a secular trend (denoting the number of observations in the entire study period), level (denoting the pre- and postintervention segments), slope (denoting the trend in the postintervention segment), time of day, and day of the week. The fit of models including linear, quadratic, and cubic trends for the postintervention segment was assessed using the AIC.

The same steps were repeated for the secondary analysis. The final model was selected in a similar way. As specified in the analytic plan, the model included terms for a secular trend, slope, month of the year, day of the week, the expert feature launch, and the national advertising campaign.

#### Unplanned Sensitivity Analyses

Prompted by inspection of the data and as a result of the review process, three unplanned sensitivity analyses (SAs) were conducted.

First, given uncertainties as to when the SARS-CoV-2 outbreak had started to affect smokers’ lives, we modelled the starting point of the outbreak as approximately 15 days before (ie, February 15, 2020) and after (ie, March 15, 2020) the original starting point (ie, March 1, 2020). These SAs were considered exploratory (as opposed to hypothesis testing).

Second, SAs were conducted to examine whether observed nonsignificant associations for the primary analysis could best be characterized as evidence of no effect or whether the data were insensitive to distinguish the null from the alternative hypothesis [18]. Bayes factors (BFs) with the alternative hypotheses conservatively represented by a half-normal distribution were calculated using an online calculator [19]. With the alternative hypothesis represented by a half-normal distribution, the standard deviation of a distribution can be specified as an expected effect size, meaning that plausible values are represented between zero and twice the effect size, with smaller values being more likely. The expected effect sizes for the level (ie, step change) and slope (ie, the trend in the postintervention period) were set as follows: first, we imagined a large step change on the basis of a hypothetical scenario in which all smokers who would otherwise attempt to quit with electronic cigarettes (e-cigarettes) or stop smoking services (ie, ~30% of the 1,890,000 UK smokers who make a quit attempt each year [20] or 567,000 smokers), spread equally across the year (ie, 567,000/365 days=1553), would switch immediately to the Smoke Free app. With a base rate of ~400 Smoke Free downloads per day, this would equate to an incidence rate ratio (IRR) of 1953/400 (IRR=4.9). Second, we imagined a large slope on the basis of the 1553 additional smokers projected to switch at a given point in time (ie, the expected large step change) instead switching gradually (and uniformly) from e-cigarettes or stop smoking services to the Smoke Free app over a 1-month period (ie, an additional 52 smokers switching per day), thus equating to a large slope or an IRR of 452/400 (IRR=1.13). Third, we imagined a small step change on the basis of a hypothetical scenario in which 5% of the 1,890,000 UK smokers who make a quit attempt each year with e-cigarettes or stop smoking services would switch immediately to the Smoke Free app (ie, 94,500/365=259). With a base rate of ~400 Smoke Free downloads per day, this would equate to an IRR of 659/400 (IRR=1.6). Fourth, we imagined a small slope on the basis of the 259 additional smokers projected to switch at a given point in time (ie, the expected small step change) instead switching gradually over a 1-month period (ie, 9 additional smokers switching per day), thus equating to an IRR of 409/400 (IRR=1.02). BFs≥3 can be interpreted as substantial evidence for the alternative hypothesis and against the null. BFs≤1/3 can be interpreted as evidence for the null hypothesis. BFs between 1/3 and 3 suggest that the data are insensitive to distinguish the alternative hypothesis from the null [18].

Third, with additional data becoming available, the primary and secondary analyses were topped up with data up to May 12, 2020. These SAs were considered exploratory.

## Results

### Analyses

**Figure 1:**
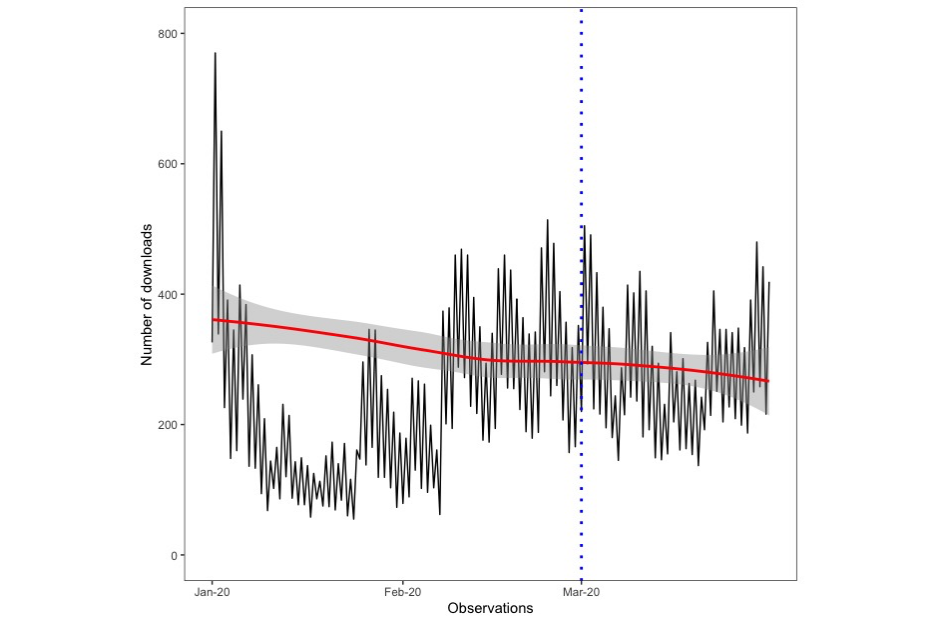
The number of users downloading the Smoke Free app from January 1, 2020, to March 31, 2020. The dotted blue vertical line represents the stipulated starting point of the severe acute respiratory syndrome coronavirus 2 outbreak (ie, March 1, 2020). The solid red line represents the fitted values with a loess smoothing function applied and associated 95% CIs.

**Figure 2:**
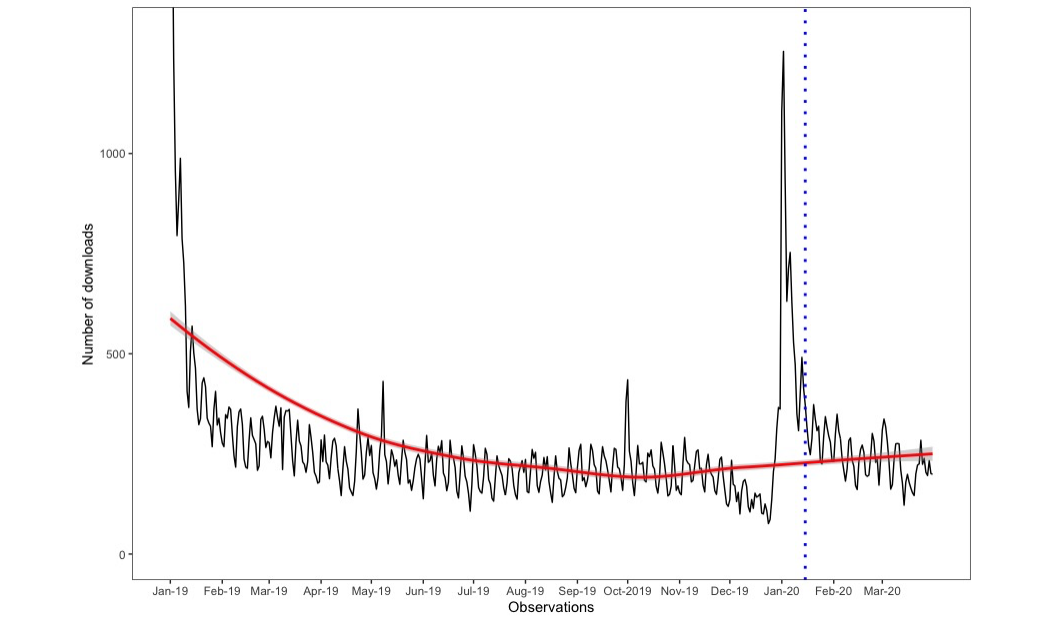
The number of users downloading the Smoke Free app from January 1, 2019, to March 31, 2020. The dotted blue vertical line represents the stipulated starting point of the severe acute respiratory syndrome coronavirus 2 outbreak (ie, January 15, 2020). The solid red line represents the fitted values with a loess smoothing function applied and associated 95% CIs. For clarity, the y-axis was capped at 1300 downloads.

Data were collected on 45,105 and 119,881 users, respectively. Figures 1 and 2 show graphs of the number of users downloading the app by month of the year for the respective analyses.

Table 2 shows the results for the best fitting segmented regression models for the primary and secondary analysis. In the primary analysis, there was no evidence for a step change or increasing trend in downloads attributable to the start of the SARS-CoV-2 outbreak on March 1, 2020. The rate of downloads was significantly greater in the evenings (IRR=1.780, 95% CI 1.581-2.004, *P*<.001). In the secondary analysis, there was evidence for a very small but significant, linearly decreasing trend in downloads over the course of the entire study period (IRR=0.996, 95% CI 0.994-0.998, *P*<.001). There was no evidence for an increasing trend in downloads attributable to the start of the SARS-CoV-2 outbreak on January 15, 2020. There was a substantial step change in downloads following the national advertising campaign (IRR=2.067, 95% CI 1.387-3.079, *P*<.001).

**Table 2:** Results from the best fitting model for each analysis.

Analysis			IRR^a^ (95% CI)		SE		*P* value		Bayes factor (small expected effect)		Bayes factor (large expected effect)
**Primary analysis: January 1, 2020-March 31, 2020^b^**											
	Trend (January 1, 2020-March 31, 2020)	0.998 (0.994-1.002)		0.002		.23		N/A^c^		N/A	
	Level (March 1, 2020)	1.142 (0.916-1.423)		0.112		.24		0.78		0.25	
	Slope (March 1, 2020-March 31, 2020)	0.999 (0.988-1.010)		0.006		.85		1.35		0.26	
	Time of day^d^	1.780 (1.581-2.004)		0.060		<.001		N/A		N/A	
**Secondary analysis: January 1, 2019-March 31, 2020^e^**											
	Trend (January 1, 2019-March 31, 2020)	0.996 (0.994-0.998)		0.001		<.001		N/A		N/A	
	Slope (January 15, 2020-March 31, 2020)	0.976 (0.908-1.049)		0.037		.51		N/A		N/A	
	Slope^2	1.001 (0.998-1.003)		0.001		.52		N/A		N/A	
	Slope^3	1.000 (1.000-1.000)		<0.001		.54		N/A		N/A	
	Expert feature launch (December 19, 2019)^f^	1.385 (0.885-2.168)		0.229		.16		N/A		N/A	
	National advertising campaign (December 27, 2019)^f^	2.067 (1.387-3.079)		0.203		<.001		N/A		N/A	

^a^IRR: incidence rate ratio.

^b^Adjusted for a second-order autoregressive term and day of the week.

^c^Not applicable.

^d^Referent=morning (vs evening).

^e^Adjusted for a first-order autoregressive term, month of the year, and day of the week.

^f^Modeled as a step change.

### Unplanned Sensitivity Analyses

#### Alternative Conceptualizations of the Outbreak

When modeling the starting point of the outbreak as February 15, 2020, and March 15, 2020, there was no evidence for a step change or increasing trend in downloads attributable to the start of the SARS-CoV-2 outbreak (see Multimedia Appendix 1).

#### Calculation of Bayes Factors

The calculation of BFs indicated that the data for the primary analysis favored the null hypothesis compared with large associations (for level, BF=0.25; for slope, BF=0.26) but were insensitive to detection of small associations (for level, BF=0.78; for slope, BF=1.35; see Table 2).

#### Analyses With Additional Data

In the SAs repeating the primary and secondary analyses with additional data up to May 12, 2020, there was no evidence for a step change or increasing trend in downloads attributable to the start of the SARS-CoV-2 outbreak (see Multimedia Appendix 1).

## Discussion

### Principal Findings

This study used an interrupted time series approach to examine the impact of the ongoing SARS-CoV-2 outbreak on the uptake of an otherwise popular smoking cessation app in UK smokers. Across two different time periods and using two different conceptualizations of the starting point of the outbreak, we found no evidence for a step change or increasing trend in the number of downloads of the popular Smoke Free app. Calculation of BFs indicated that the data favored the null compared with large associations but were insensitive to detection of small associations.

### Limitations

First, the small number of observations since the start of the SARS-CoV-2 outbreak means that we likely had low statistical power to detect anything but large effects. Second, pinpointing the exact starting point of the outbreak was not straightforward, as it is unclear when UK smokers first became aware of or affected by the pandemic, hypothesized to act as triggers for additional quit attempts. This was partly mitigated by conducting two SAs, which also did not provide evidence for a step change or increasing trend in downloads. Third, given the current structure of the app’s database, we were unable to distinguish between downloads of the free and “pro” versions of the Smoke Free app in our analyses. Although a tally is kept for free and “pro” downloads combined, the tally kept for “pro” accounts has been designed to track weekly subscriptions (with each user counted several times) as opposed to one-off downloads. It is plausible that a greater number of smokers who otherwise would have tried to stop using e-cigarettes or stop smoking services would now be more inclined to purchase the “pro” version of the app. Fourth, the Smoke Free app is only one of many available online stop smoking platforms in the United Kingdom. We did not capture traffic on other available apps or websites such as the NHS Smokefree website [21] and were, hence, unable to model any effect of the SARS-CoV-2 outbreak on interest in stop smoking support (digital or nondigital) more broadly. For example, although vape shops are temporarily closed, it is plausible that online sales of e-cigarettes have increased during the study period. Fifth, we did not take account of tobacco industry behavior in our models. There has also been legitimate scientific investigation into the possible links between smoking, nicotine, and SARS-CoV-2 infection and COVID-19 outcomes [22,23]. In the absence of clear evidence as to whether smokers are at increased or reduced risk, it is plausible that the tobacco industry has attempted to capitalize on this uncertainty by, for example, influencing media reports, which may in turn have influenced quitting behavior.

### Implications for Policy

The lack of an uptick in downloads of a popular smoking cessation app during the SARS-CoV-2 outbreak thus far suggests that smokers may not be turning to available, evidence-informed digital support in greater numbers. Evidence from controlled studies and population-level surveys indicate that smoking cessation attempts involving pharmacological or behavioral support (including digital interventions) are substantially more likely to be successful compared with unassisted attempts [24-26]. In the absence of readily available alternatives due to social distancing measures, UK public health bodies should consider putting resources toward increasing awareness of available, evidence-informed mobile health support.

### Conclusions

In the UK, between January 1, 2020, and March 31, 2020, and between January 1, 2019, and March 31, 2020, there was no evidence that the SARS-CoV-2 outbreak thus far has been associated with a large step change or increasing trend in downloads of a popular smoking cessation app. Findings on the association of the SARS-CoV-2 outbreak with a small step change or increasing trend were inconclusive.

## Appendix

Multimedia Appendix 1 Sensitivity analyses.

## Abbreviations

AIC: Akaike information criterion
AR: autoregressive
BF: Bayes factor
COVID-19: coronavirus disease
e-cigarettes: electronic cigarettes
GAMM: generalized additive mixed model
IRR: incidence rate ratio
MA: moving average
SA: sensitivity analysis
SARS-CoV-2: severe acute respiratory syndrome coronavirus 2
