# Supplementary material for: Influence of the SARS-CoV-2 Outbreak on the Uptake of a Popular Smoking Cessation App in UK Smokers: Interrupted Time Series Analysis
Source: JMIR Mhealth Uhealth. 2020 Jun 11;8(6):e19494. doi: 10.2196/19494 (PMC7296974; doi:10.2196/19494)
Supplement: Multimedia Appendix 1 [file mhealth_v8i6e19494_app1.docx]

**Multimedia Appendix 1**

**Sensitivity Analyses**

*Table.* Results from the best fitting model for the two sensitivity analyses (SAs) with the starting point of the outbreak modelled as 15 February 2020 and 15 March 2020, respectively.

|  | IRR^a^ (95% CI) | *SE* | *P* |
| --- | --- | --- | --- |
| **SA1: 1 January 2020 to 31 March 2020**^b^ |  |  |  |
| Trend | 0.995 (0.989, 1.001) | 0.003 | .09 |
| Level (15 February 2020) | 1.062 (0.811, 1.391) | 0.138 | .66 |
| Slope | 1.010 (0.989, 1.032) | 0.011 | .36 |
| Slope^2 | 0.9999 (0.9997, 1.000) | < 0.001 | .54 |
| Time of day^c^ | 1.808 (1.616, 2.023) | 0.057 | < .001 |
|  |  |  |  |
| **SA2: 1 January 2020 to 31 March 2020**^b^ |  |  |  |
| Trend | 0.998 (0.995, 1.001) | 0.002 | .14 |
| Level (15 March 2020) | 0.964 (0.751, 1.237) | 0.127 | .77 |
| Slope | 1.007 (0.989, 1.025) | 0.009 | .45 |
| Time of day^c^ | 1.799 (1.604, 2.018) | 0.059 | < .001 |

*Note.* ^a^ IRR = incidence rate ratio; ^b^ Adjusted for AR(2) and day of the week; ^c^ Referent = morning (vs. evening).

*Table.* Results from the best fitting model for the two sensitivity analyses (SAs) with additional data up to 12 May 2020.

|  | IRR^a^ (95% CI) | *SE* | *P* |
| --- | --- | --- | --- |
| **SA1: 1 January 2020 to 12 May 2020**^b^ |  |  |  |
| Trend (1 January 2020 to 12 May 2020) | 0.998 (0.995, 1.001) | 0.002 | .23 |
| Level (1 March 2020) | 1.109 (0.928, 1.326) | 0.091 | .26 |
| Slope (1 March 2020 to 12 May 2020) | 0.998 (0.992, 1.003) | 0.003 | .34 |
| Time of day^c^ | 1.691 (1.519, 1.882) | 0.055 | < .001 |
|  |  |  |  |
| **SA2: 1 January 2019 to 12 May 2020**^d^ |  |  |  |
| Trend (1 January 2019 to 12 May 2020) | 0.996 (0.994, 0.997) | 0.001 | < .001 |
| Slope (15 January 2020 to 12 May 2020) | 0.990 (0.948, 1.035) | 0.022 | .66 |
| Slope^2 | 1.000 (0.999, 1.001) | 0.001 | .71 |
| Slope^3 | 1.000 (1.000, 1.000) | < 0.001 | .75 |
| Expert feature launch (19 December 2019)^e^ | 1.391 (0.896, 2.158) | 0.224 | .14 |
| National advertising campaign (27 December 2019)^e^ | 2.076 (1.405, 3.068) | 0.199 | < .001 |

*Note.* ^a^ IRR = incidence rate ratio; ^b^ Adjusted for AR(2) and day of the week; ^c^ Referent = morning (vs. evening); ^d^ Adjusted for AR(1), month of the year and day of the week; ^e^ Modelled as a step change.
